# A Challenging Case of Acute Mercury Toxicity

**DOI:** 10.1155/2018/1010678

**Published:** 2018-02-18

**Authors:** Ali Nayfeh, Thamer Kassim, Noor Addasi, Faysal Alghoula, Christopher Holewinski, Zachary Depew

**Affiliations:** ^1^Department of Internal Medicine, Creighton University, Omaha, NE, USA; ^2^Catholic Health Initiative (CHI), Englewood, CO, USA; ^3^Division of Pulmonary, Critical Care, and Sleep Medicine, Creighton University, Omaha, NE, USA

## Abstract

**Background:**

Mercury exists in multiple forms: elemental, organic, and inorganic. Its toxic manifestations depend on the type and magnitude of exposure. The role of colonoscopic decompression in acute mercury toxicity is still unclear. We present a case of acute elemental mercury toxicity secondary to mercury ingestion, which markedly improved with colonoscopic decompression.

**Clinical Case:**

A 54-year-old male presented to the ED five days after ingesting five ounces (148 cubic centimeters) of elemental mercury. Examination was only significant for a distended abdomen. Labs showed elevated serum and urine mercury levels. An abdominal radiograph showed radiopaque material throughout the colon. Succimer and laxatives were initiated. The patient had recurrent bowel movements, and serial radiographs showed interval decrease of mercury in the descending colon with interval increase in the cecum and ascending colon. Colonoscopic decompression was done successfully. The colon was evacuated, and a repeat radiograph showed decreased hyperdense material in the colon. Three months later, a repeat radiograph showed no hyperdense material in the colon.

**Conclusion:**

Ingested elemental mercury can be retained in the colon. Although there are no established guidelines for colonoscopic decompression, our patient showed significant improvement. We believe further studies on this subject are needed to guide management practices.

## 1. Introduction

Mercury exists in multiple forms: elemental, organic, and inorganic. Its toxic manifestations depend on the type and magnitude of exposure which can range from a minor to a life-threatening presentation. In this article, we present a case of mercury toxicity due to elemental mercury ingestion. We discuss the human use of elemental mercury and its exposure, routes of absorption, and clinical manifestations of its toxicity. We will also discuss the assessment and management of elemental mercury toxicity.

## 2. Case Presentation

A 54-year-old male with past medical history of degenerative joint disease, major depressive disorder, polysubstance dependence, and history of childhood burn presented to the Emergency Department (ED) complaining of imbalance, irritability, outburst of temper, fatigue, and weakness for the last five days after he ingested five ounces (oz) (148 cc) of mercury as a suicidal attempt. In the ED, the patient denied any changes in vision, hearing impairment, or tremors. He also denied shortness of breath, abdominal pain, nausea, vomiting, or diarrhea.

On physical examination, the patient appeared to be in no obvious distress. He seemed well developed, alert, and oriented to time, place, and person. Vital signs (BP 144/96, HR 74, RR 16, and temperature 96.3). A neurological examination yielded intact cranial nerves, with normal motor function and no sensory deficits. Abdominal examination showed a distended and nontender abdomen with normoactive bowel sounds. The rest of the physical examination showed no abnormalities.

**Figure 1:**
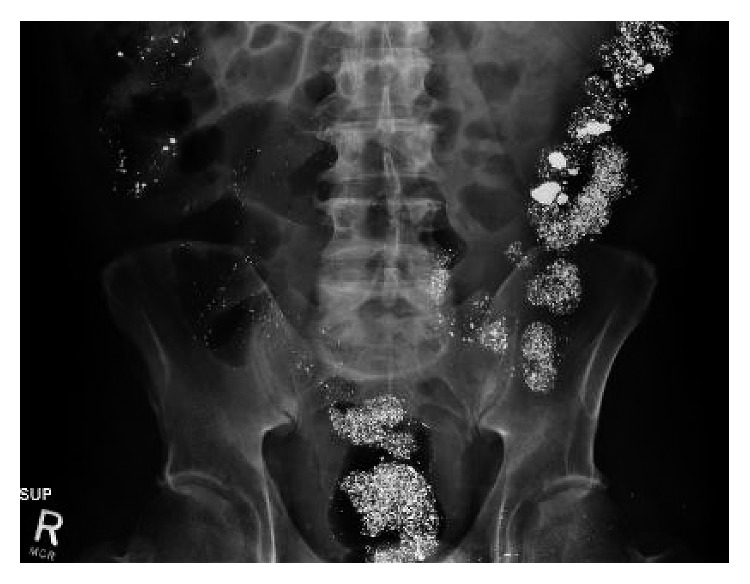


On admission, laboratory workup was done and showed hemoglobin of 13.9 gm/dl (normal range 13.5–17.5), platelet of 309 k/ul (normal range is 40–440), WBC of 8 k/ul (normal range 4–12), a mildly elevated creatinine at 1.4 mg/dl (eGFR 56) (reference range 0.6–1.3 mg/dl), and a low potassium at 3.5 (reference range 3.7–5.1 mg/dl). A urine drug screen was done which was positive for amphetamine, benzodiazepine, and cannabinoid. A mercury toxicology workup was initiated and showed an elevation in serum mercury level at 110 *µ*g/l (reference range < 10 *µ*g/l), urine mercury level at 37 *µ*g/l (reference range < 10 *µ*g/l), and 24-hour urinary mercury level at 248 *µ*g (no exposure < 20 *µ*g/24, inconclusive 20–150 *µ*g/24h, and potentially toxic > 150 *µ*g/24h). Initial abdominal X-ray (Figure 1) showed diffuse radiopaque material visualized throughout the colon.

Due to the patient's toxic mercury levels and worrying neurological signs and symptoms, the patient was admitted to the intensive care unit with a one on one sitter available at his bedside. Vital signs, neurological checks, and electrolytes were measured on regular basis. The poison-control team was consulted upon admission and recommended starting the patient on succimer, as a chelating agent, to be given in a dose of 500 mg every eight hours for the first 5 days then every twelve hours for the 14 days. The patient was also started on polyethylene glycol 17 gm twice a day and magnesium citrate to enhance GI motility along with intravenous fluids and continuous replacement of electrolytes. The neurology team was consulted for further evaluation of the patient neurological complaints. They agreed with the current plan of care; head computed tomography (CT) was performed and showed no intracranial abnormalities.

The psychiatry team was asked to assess the patient for his suicidal attempt and was evaluated as nonsuicidal at that time and was started on escitalopram 10 mg. Bedside sitter was discontinued.

The patient was showing slow progress in term of feeling weak, fatigue, and imbalance during his stay but remained hemodynamically stable. The patient was transferred out of the ICU to the floor, and serial abdomen X-rays were done. The patient was having recurrent bowel movements on daily basis, and X-rays showed continued decrease in the amount of mercury in the descending colon with interval increase in the hyperdense materiel in the cecum and ascending colon. Mercury levels were trended during the patient's hospitalization, and results are shown in Table 1.

**Figure 2:**
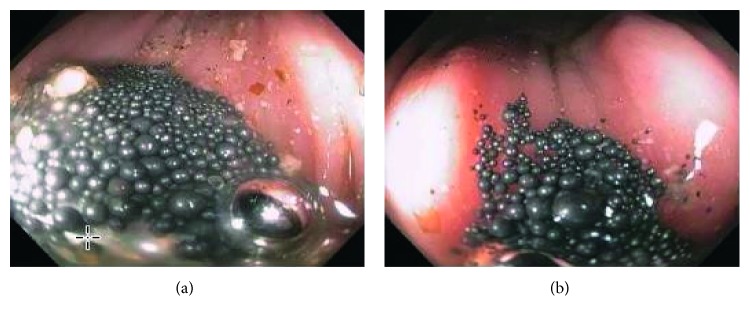
(a) Cecum. (b) Ascending colon.

**Figure 3:**
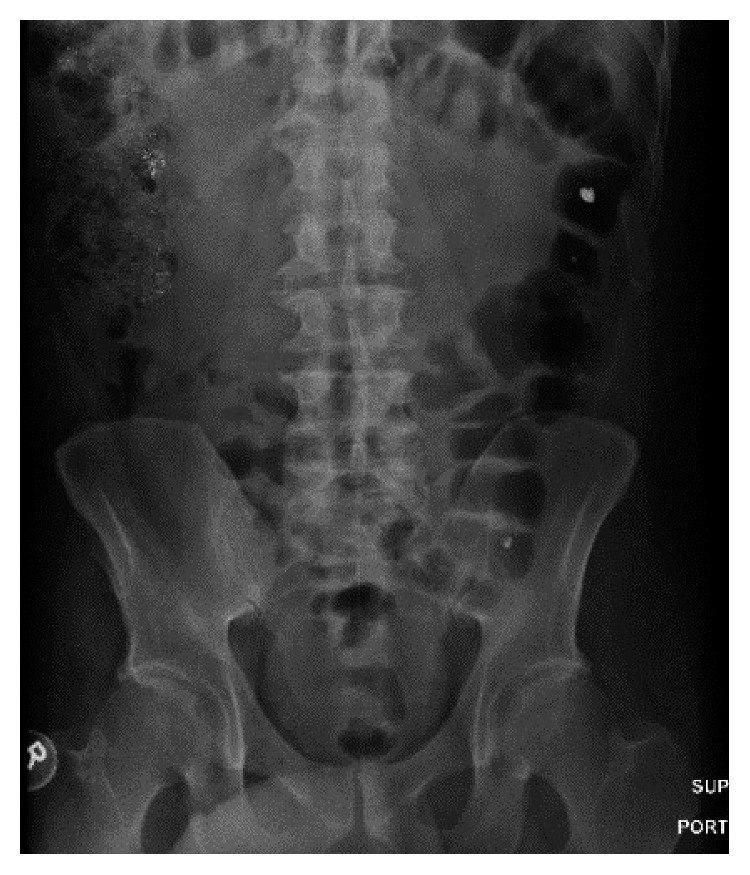


**Figure 4:**
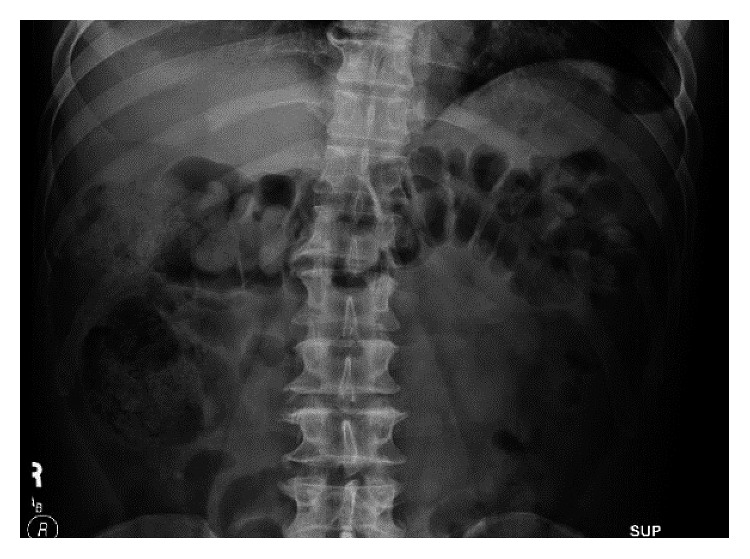


**Figure 5:**
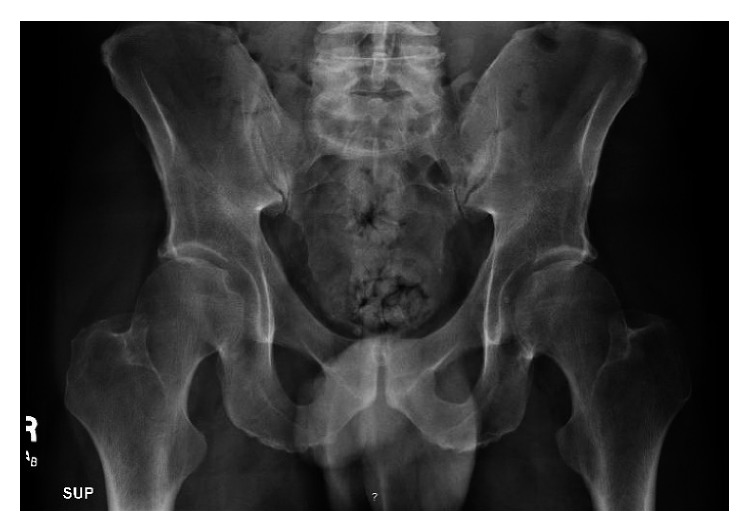


The gastroenterology team was consulted and recommended placing a nasogastric tube and to give 4 liters of polyethylene glycol through the tube and magnesium citrate every 8 hours in an attempt to enhance gastrointestinal motility and hasten mercury clearance. Repeated X-rays showed no advancement of the hyperdense material which remained in the cecum and ascending colon. The decision was made to do a colonoscopy for an attempt of colonoscopic decompression (Figure 2). The colon was evacuated with copious amount of washing, and a repeat abdominal X-ray showed decreased hyperdense material in the colon with a nonobstructive gas pattern (Figure 3). The patient was discharged in a good condition after a 10-day hospital stay and followed up after 3 months; at that time, he had a mercury level of 33, and an abdominal X-ray showed no hyperdense material in the colon (Figures 4 and 5). The patient followed up again 1 month later. His neurological symptoms resolved, and kidney function preserved. Also, his mercury level was trending down at 18.

## 3. Discussion

### 3.1. Brief Introduction, Sources, and Exposure

Elemental mercury is a silver-colored liquid that is volatile at room temperature and causes pulmonary, neurological, and renal toxicity [1]. It is used in many technical and medical instruments including sphygmomanometers, manometers, thermometers, barometers, and compact fluorescent light bulbs as well [2, 3]. Mercury has been used widely in amalgam dental filling, which can also be a source of elemental mercury exposure to patients, dental technicians, and dental practitioners. However, studies have not correlated any symptoms or clinically significant health effects with absorption from dental amalgams [2]. Other sources of exposure can be from ingestion of herbal medications for vertigo management, inhalation of mercury vapor for pain relief of arthralgia, and inhalation of mercury vapor for hemorrhoid treatment [2].

### 3.2. Absorption

The major route of absorption is by diffusion through the respiratory tract where up to 80% of inhaled mercury vapor is expected to diffuse into the bloodstream. Absorption from the gastrointestinal tract is very poor with a bioavailability of less than 0.01% and most of the ingested mercury is eliminated in feces [2]. Absorption through the skin is limited as well [2]. Although ingestion of elemental mercury is poorly absorbed through the gastrointestinal tract, mercury globules in the gastrointestinal tract can release vapor at body temperature and this can be absorbed by the lungs. Moreover, the metal can be absorbed more readily in cases of diverticulitis or GI abscesses where there is intense inflammation and mucosal breakdown causing increased bioavailability and systemic manifestations. Also in cases of diverticulosis and GI abscesses, there is a possibility of conversion of elemental mercury to the organic form by bacteria, leading to systemic toxicities [2, 4].

### 3.3. Distribution and Metabolism

The oral lethal dose 10 is approximately 100 grams for a 70 kg adult [5]. In our case, the patient ingested 5 ounces of elemental mercury which is equivalent to around 141.75 grams causing a higher level of absorption. Also, the patient presented five days after ingestion of elemental mercury which we believe increased the chance of the metal to volatilize and diffuse into the bloodstream. This was demonstrated by the patient's neurological symptoms and the high levels of blood and urine mercury.

### 3.4. Clinical Manifestations (Acute and Chronic), Diagnosis, and Management

The clinical manifestations depend on the magnitude of elemental mercury exposure and whether it was acute or chronic. Acute toxicity can be seen in the setting of industrial exposure in which the inhalation of mercury vapor results in hypoxia, permanent lung damage, and even death (2). Inhaled mercury vapor can cause several neurological manifestations due to its ability to diffuse through the blood brain barrier. These manifestations can be reversible once the metal is cleared out of the body and include tremors, paresthesia, memory loss, hyperexcitability, and delayed reflexes [2, 6]. Other symptoms occurring with mercury toxicity include cough, dyspnea, stomatitis, excessive salivation, nausea, vomiting, diarrhea, conjunctivitis, and dermatitis.

With chronic exposure to elemental mercury, central nervous system and kidneys are the main affected organs. The major clinical features of chronic mercury poisoning include tremors, psychological disturbances, erethism, and gingivitis [2, 7]. Tremor, either intentional or resting, is considered to be an early neurological sign of poisoning. Erethism is the hallmark of mercury poisoning where features include a change in personality, anxiety, excitability, fearfulness, pathologic shyness along with insomnia, memory loss, depression, fatigue, and outbursts of temper. Proteinuria is the most common sign of kidney effects due to tubular damage. Nephrotic syndrome can occur in severe cases. In addition, peripheral nerve abnormalities can occur but are not very common [2].

Diagnosing elemental mercury toxicity depends on exposure history and clinical manifestations. Blood mercury level is a useful test especially if the level of exposure was high (2). However, once absorbed into the blood, the serum half-life is relatively short lasting for approximately three days as the level deceases due to redistribution in the body [8]. The overall half-life of mercury in the body is approximately 1–3 months [8]. The 24-hour urine mercury level is the best biomarker for chronic long-term exposure. Chest X-ray may be needed in the case of presence of respiratory symptoms especially with inhalational toxicity. Abdominal X-ray may show the deposition of mercury in the GI tract in cases of oral ingestion as in our patient.

When managing cases of mercury toxicity, treatment starts with eliminating the source of exposure and initiating supportive measures including oxygen, bronchodilators, and fluid resuscitation. Lowering the body level concentration is fundamental in selected cases by using chelating agents especially if the blood and urine concentrations are above 100 mcg/L [9]. (Chelating agents increase the urinary excretion of mercury [9] which includes thiol-based agents such as dimercaprol (British anti-Lewisite (BAL)), penicillamine, unithiol (2,3-dimercaptopropane-1-sulfonate (DMPS)), and succimer (dimercaptosuccinic acid (DMSA)).

Retained mercury in the colon can be removed using agents that increase GI motility and colonoscopy. Although the role of colonoscopy is not an established evidence-based recommendation, there are previously reported cases of colonoscopic decompression and evacuation [10].

## 4. Conclusion

Ingestion of elemental mercury can be retained in the colon. Vigorous GI cleansing via motility enhancing medications and colonoscopy can be used to hasten elimination. The role of colonoscopic decompression in elemental mercury ingestion is not established, but there are previously reported cases where colonoscopy was used for evacuation.

## Figures and Tables

**Table 1:** 

	Admission	Day 2	Day 4	Day 6	3 months after discharge	4 months after discharge
Serum mercury level (*µ*g/l)	110	134	122	N/A	33	18
24-hour urine mercury	248	499	N/A	233	N/A	N/A

Serum mercury reference range < 10 *µ*g/l; 24-hour urine mercury reference range: nonexposure < 20 *µ*g/24 h; inconclusive 20 to 150 *µ*g/24 h; potentially toxic > 150 *µ*g/24 h.
